# An Endurance-Dominated Exercise Program Improves Maximum Oxygen Consumption, Ground Reaction Forces, and Muscle Activities in Patients With Moderate Diabetic Neuropathy

**DOI:** 10.3389/fphys.2021.654755

**Published:** 2021-03-18

**Authors:** AmirAli Jafarnezhadgero, Elahe Mamashli, Urs Granacher

**Affiliations:** ^1^Department of Sport Management and Biomechanics, University of Mohaghegh Ardabili, Ardabil, Iran; ^2^Division of Training and Movement Sciences, Research Focus Cognition Sciences, University of Potsdam, Potsdam, Germany

**Keywords:** oxygen consumption, kinetics, electromyography, diabetic, gait

## Abstract

**Background:**

The prevalence of diabetes worldwide is predicted to increase from 2.8% in 2000 to 4.4% in 2030. Diabetic neuropathy (DN) is associated with damage to nerve glial cells, their axons, and endothelial cells leading to impaired function and mobility.

**Objective:**

We aimed to examine the effects of an endurance-dominated exercise program on maximum oxygen consumption (VO_2_max), ground reaction forces, and muscle activities during walking in patients with moderate DN.

**Methods:**

Sixty male and female individuals aged 45–65 years with DN were randomly assigned to an intervention (IG, *n* = 30) or a waiting control (CON, *n* = 30) group. The research protocol of this study was registered with the Local Clinical Trial Organization (IRCT20200201046326N1). IG conducted an endurance-dominated exercise program including exercises on a bike ergometer and gait therapy. The progressive intervention program lasted 12 weeks with three sessions per week, each 40–55 min. CON received the same treatment as IG after the post-tests. Pre- and post-training, VO_2_max was tested during a graded exercise test using spiroergometry. In addition, ground reaction forces and lower limbs muscle activities were recorded while walking at a constant speed of ∼1 m/s.

**Results:**

No statistically significant baseline between group differences was observed for all analyzed variables. Significant group-by-time interactions were found for VO_2_max (*p* < 0.001; *d* = 1.22). The *post-hoc* test revealed a significant increase in IG (*p* < 0.001; *d* = 1.88) but not CON. Significant group-by-time interactions were observed for peak lateral and vertical ground reaction forces during heel contact and peak vertical ground reaction force during push-off (*p* = 0.001–0.037; *d* = 0.56–1.53). For IG, *post-hoc* analyses showed decreases in peak lateral (*p* < 0.001; *d* = 1.33) and vertical (*p* = 0.004; *d* = 0.55) ground reaction forces during heel contact and increases in peak vertical ground reaction force during push-off (*p* < 0.001; *d* = 0.92). In terms of muscle activity, significant group-by-time interactions were found for vastus lateralis and gluteus medius during the loading phase and for vastus medialis during the mid-stance phase, and gastrocnemius medialis during the push-off phase (*p* = 0.001–0.044; *d* = 0.54–0.81). *Post-hoc* tests indicated significant intervention-related increases in vastus lateralis (*p* = 0.001; *d* = 1.08) and gluteus medius (*p* = 0.008; *d* = 0.67) during the loading phase and vastus medialis activity during mid-stance (*p* = 0.001; *d* = 0.86). In addition, *post-hoc* tests showed decreases in gastrocnemius medialis during the push-off phase in IG only (*p* < 0.001; *d* = 1.28).

**Conclusions:**

This study demonstrated that an endurance-dominated exercise program has the potential to improve VO_2_max and diabetes-related abnormal gait in patients with DN. The observed decreases in peak vertical ground reaction force during the heel contact of walking could be due to increased vastus lateralis and gluteus medius activities during the loading phase. Accordingly, we recommend to implement endurance-dominated exercise programs in type 2 diabetic patients because it is feasible, safe and effective by improving aerobic capacity and gait characteristics.

## Introduction

The prevalence of diabetes worldwide is predicted to increase from 2.8 to 4.4% between 2000 and 2030 (Wild et al., 2004). In over 50% of diabetic neuropathy (DN) patients, substantial, irreparable nerve damage already occurs before diagnosis, making this condition the leading cause of diabetes-related hospital admissions and non-traumatic amputations worldwide (Richner et al., 2018). More specifically, DN is associated with damage to nerve glial cells, their axons, and endothelial cells leading to impaired function and mobility (Nozabieli et al., 2014; Peltier et al., 2014; Richner et al., 2018). Mueller et al. (1994) reported that DN individuals aged 35–75 years walk with ∼15% slower speed compared to age matched healthy controls. Moreover, Akashi et al. (2008) evaluated differences in ground reaction forces during walking in DN patients compared with healthy controls aged 40–70 years. These authors observed a 3.6% lower second peak of vertical ground reaction force in DN individuals. Kwon et al. (2003) examined neuromuscular activity during walking in DN patients aged 40–70 years and found premature activation of soleus and medial gastrocnemius muscles which appears to contribute to abnormal forefoot plantar pressure distribution in DN individuals (Kwon et al., 2003). Furthermore, premature activation of the medial gastrocnemius together with a prolonged tibialis anterior activity results in muscle co-activation during mid-stance and seems to be a compensatory mechanism to enhance joint stability (Sacco and Amadio, 2003). Sawacha et al. (2012) examined lower limbs muscle activities while walking in DN patients aged 61 years and found a delay in gluteus medius activity during the terminal swing phase in DN patients compared with controls (Sawacha et al., 2012). During walking, the gluteus medius acts as a hip abductor to stabilize the pelvis as the contralateral leg swings through (Perry and Davids, 1992). Weakness of the gluteus medius may result in adverse changes in kinematics (French et al., 2010) and a concomitant increase risk of injury (Leetun et al., 2004; Stastny et al., 2016). Besides diabetes-related neural degeneration with subsequent mobility limitations, there is evidence for declines in aerobic capacity in DN patients. In fact, Regensteiner et al. (1998) demonstrated a ∼29% lower maximum oxygen consumption (VO_2_max) in DN patients compared with healthy controls aged 30–50 years.

Due to the rather high DN prevalence rates and the disease-related symptoms and functional limitations, rehabilitation programs are needed which have the potential to effectively treat DN. Previous studies (Weintraub et al., 2009; Streckmann et al., 2014; Waldfogel et al., 2017; Amato Nesbit et al., 2019; Zhang and Liu, 2019) examined the effects of pharmacological drugs, manual therapy, electrotherapy, acupuncture, and exercise therapy. Particularly exercise therapy appears to be effective because it has the potential to improve patients’ aerobic capacity (e.g., VO_2_max) (Rehman et al., 2017) and their gait characteristics (Allet et al., 2010; El-Refay and Ali, 2013; Suzuki et al., 2019). Rehman et al. (2017) examined the impact of a 25 week endurance training program with three sessions per week on VO_2_max in male and female DN patients aged 40–70 years. The endurance training was performed on a treadmill with progressive increments in inclination starting from zero degree during week 1 and ending with 12 degree during week 25. Compared with a control group, the intervention group showed a statistically significant 8.6% increase in VO_2_max after training (Rehman et al., 2017). In another study, Tsang et al. (2007) investigated the effects of a 16 week Tai Chi program with two sessions per week on measures of walking speed and static/dynamic balance using a balance test battery (i.e., Chattecx Balance System) in diabetic patients aged >50 years. Compared with a control group, the exercise group showed statistically significant improvements in measures of walking speed (2.1%) and balance (3.4–28.2%). Goryachev et al. (2011) investigated the effects of a 3 months gait therapy program on measures of lower limbs muscle activities during walking in patients with knee osteoarthritis aged ∼60 years. Compared with a control group, the exercise group showed statistically significant increases vastus lateralis (71%) and tibialis anterior (79%) activities (Goryachev et al., 2011). Finally, a recent systematic review of randomized controlled trials evaluated the impact of multimodal exercise therapy on gait function in DN patients (Melese et al., 2020). Based on the outcomes of eight studies, this review suggests that multimodal exercise programs consisting of strength, balance, stretching exercises, and gait training significantly improved gait function (e.g., walking speed, cadence, and stride length) in DN patients compared with a control group.

Less is known on the underlying physiological and biomechanical mechanisms responsible for the observed exercise induced adaptations in DN patients. Therefore, in an attempt to fill this void in the literature, we investigated the effects of an endurance-dominated exercise program on changes in VO_2_max, ground reaction forces, and lower limbs muscle activity during walking in individuals with DN. Of note, the endurance-dominated exercise program included training on a bike ergometer together with gait therapy. This program might be particularly effective because it has the potential to improve both, aerobic capacity and markers of gait stability. With reference to the relevant literature (Goryachev et al., 2011; Rehman et al., 2017; Melese et al., 2020), we hypothesized that an endurance-dominated exercise program in combination with gait therapy results in enhanced VO_2_max and reduced peak vertical ground reaction forces and concomitant increases in vastus lateralis activity during walking in DN patients.

## Materials and Methods

### Study Design and Participants

**FIGURE 1:**
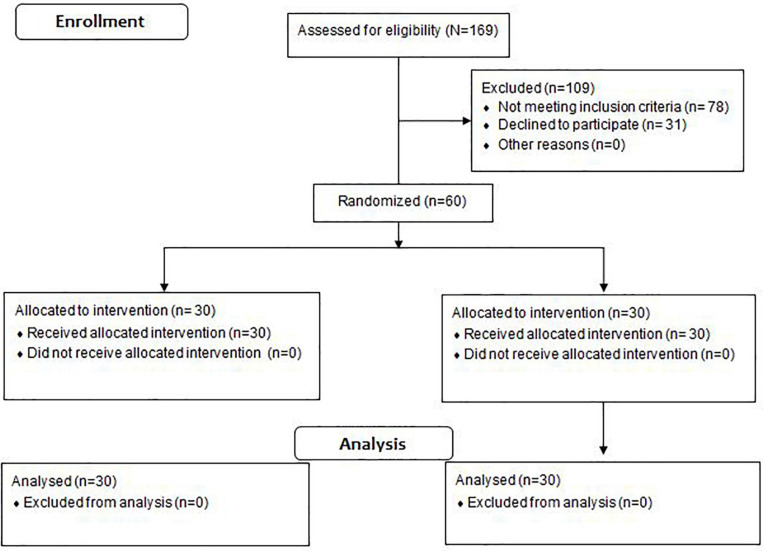
Flow diagram of the randomized controlled trial.

We used the freeware tool GPower^1^ to calculate a one-sided *a priori* power analysis. The power analysis was computed using the *F*-test family (i.e., ANOVA repeated measures within-between interaction) and a related study that examined the effects of aerobic training on VO_2_max in DN patients (Najafipour et al., 2017). The included program variables were an assumed Type I error of 0.05, a Type II error rate of 0.20 (80% statistical power), and an effect size of 0.80 (i.e., interaction effects) for VO_2_max. The analysis revealed that at least 15 participants would be needed per group to achieve large-sized interaction effects for VO_2_max. Sixty male and female individuals aged 45–65 years with a history of diabetes >10 years and diagnosed symptoms of neuropathy volunteered to participate in this study (Figure 1). The participants were randomly assigned to an intervention (IG, *n* = 30; females = 10, males = 20) or a control group (CON, *n* = 30; females = 10, males = 20). The block randomization method (block size = 4) was used to allocate study participants into experimental groups (Lachin et al., 1988; Frane, 1998). A naïve examiner realized the block randomization process. During the randomization procedure, a set of sealed, opaque envelopes was used to ensure the concealment of the allocation. Each envelope contained a card stipulating to which group the participant would be allocated to. Of note, participants were blinded to group allocation. One examiner determined whether a participant was eligible for inclusion, while the other carried out gait analyses of the eligible participants. Both examiners were unaware of group allocation. Another naïve examiner (i.e., physiotherapist with 10 years professional experience) controlled allocation of each participant and was responsible for delivering the treatment to both groups.

Ethical approval was obtained from the local ethical committee (IR.ARUMS.REC.1397.287). The research protocol of this study was registered with the Iranian Clinical Trial Organization (IRCT20200201046326N1). All participants provided their written informed consent prior to the start of the study. This study was designed and conducted as a double-blinded randomized-controlled-trial (i.e., participants, examiners) (Figure 1). Accordingly, we followed the CONSORT-Statement which is illustrated in the Supplementary File (Appendix 1). Experimental group allocation was matched according to DN severity (Picon et al., 2012), age, and body mass index. The severity of DN was determined in accordance with the fuzzy scoring system (Picon et al., 2012). The model variables of the fuzzy scoring system were used to classify neuropathy in diabetic patients as mild, moderate, or severe (Picon et al., 2012). More specifically, symptoms were assessed through the Michigan Neuropathy Screening Instrument questionnaire score, level of hemoglobin A1c (HbA1c), and the time span in years after first DN diagnosis. The Michigan Neuropathy Screening Instrument (MNSI) questionnaire is self-administered. All responses add up to a final score. Each participant’s “yes” response to the questions 1–3, 5–6, 8–9, 11–12, and 14–15 were counted as one point. In addition, each participant’s “no” response to the questions 7 and 13 were counted as one point. Question 4 was considered to be a measure of impaired circulation and question 10 a measure of general asthenia. Accordingly, these questions were not included in the published scoring algorithm (Feldman et al., 1994). During the MNSI examination, a health professional inspected each foot for deformities, dry skin, calluses, infections and fissures. Each foot with any abnormality received a score of 1. Each foot was also inspected for ulcers and each foot with an ulcer received a score of 1. Moreover, ankle reflexes were elicited. If the reflex was absent, the patient was asked to perform the Jendrassik maneuver and, if present, the reflex was deemed “functioning” with reinforcement and was scored as 0.5. If the reflex was absent with the Jendrassik maneuver, the reflex was deemed “absent” and scored as 1. Vibration sensation was then tested in the great toe using a 128-Hz tuning fork. In general, the examiner should be able to sense vibration in his or her hand for 5 s longer than a normal subject can at the great toe. Vibration is scored as present if the examiner senses the vibration on his or her finger for <10 s longer than the subject feels it in the great toe, decreased if sensed for ≥10 s (scored as 0.5) or absent (scored as 1). The overall score amounts to eight points and, in the published scoring algorithm, a score ≥2.5 is considered abnormal (Feldman et al., 1994). In sum, the following criteria were adopted for patients to be included; (i) a score between 4.6 and 7.5 in the fuzzy scoring system; (ii) at least 10 years after first type 2 diabetes diagnosis; (iii) age under 65 years; (iv) no foot ulcers during the study period.

Participants were excluded if they (i) suffered partial or total lower limb amputation or other neurological or orthopedic impairments due to stroke, cerebral palsy, poliomyelitis, rheumatoid arthritis, prosthesis, or moderate or severe osteoarthritis; (ii) regularly performed exercise during the last 6 months; (iii) were unable to walk independently without pain or the use of an assistive device; (iv) had serious cardiac pathology, unstable hypertension, or serious musculoskeletal problems that would limit their ability to exercise.

Body height was measured to the nearest 1 mm using a wall mounted stadiometer. For waist circumference, the measurement was taken at the approximate midpoint between the lower margin of the last palpable rib and the top of the iliac crest (World Health Organization, 2011). Hip circumference was taken around the widest portion of the buttocks (i.e., parallel to the floor at the level at which the measurement was taken). Bioimpedance analysis (BIA) was conducted for the assessment of body mass and body fat using a Tanita BIA body fat analyzer (TBF-401, Tanita Co., Tokyo, Japan) during the morning hours (Sung et al., 2001). Participants were asked to stand barefoot on the metal sole plates of the testing instrument. Sex and body height were entered manually into the system via a keyboard. Body mass and percentage body fat were displayed on the BIA machine and printed out for further analysis.

### Endurance-Dominated Exercise Program

Over a period of 12 weeks, an endurance-dominated exercise program was conducted that included progressive training on a bike ergometer and gait therapy [i.e., continuous walking, stair climbing and descending, tandem walking, and walking sideways (Ahmad et al., 2020)]. Three training sessions were scheduled per week and each lasted between 40 and 55 min. The contents of the program were in accordance with physical activity recommendations of the American Diabetes Association (Table 1). Before the start of the study, training intensity on the bike ergometer was individually determined using a graded exercise protocol on a bike ergometer and VO_2_max as outcome measure. During the first week of intervention, training intensity was set at 55% of VO_2_max and was progressively increased until 75% over the course of the intervention. All training sessions were supervised by health care professionals and delivered in the gym of a diabetes hospital. Blood pressure, blood glucose levels, and heart rate were regularly checked before each exercise session. Participants were not permitted to exercise if the resting blood pressure was greater than 200 mm Hg (systolic) or greater than 110 mm Hg (diastolic). Similar considerations were made regarding the respective blood glucose level which was in accordance with recommendations of the American Diabetes Association (Association and American Diabetes, 2014). If patients’ blood glucose levels were 100–250 mg/dL, the exercise program was prescribed. The exercise program was supervised by a physiotherapist with 10 years professional experience to ensure that there was no contraindication to exercise. To be allowed to participate in the exercise program, the cardiologist had to provide permission after a physical examination. In addition, a visual foot examination (deformities, dry skin, calluses, infections, and fissures) was performed by health care professionals (i.e., neurophysiologist and a podiatrist with ∼10 years of professional experience) once weekly to ensure the absence of foot ulcer development. Moreover, participants were encouraged to inspect their feet daily. Gait therapy included continuous walking, stair climbing and descending, tandem walking and walking sideways (Table 1; Ahmad et al., 2020). Exercises were incorporated into gait therapy if they targeted muscles that are specifically prone to weakness (e.g., medial gastrocnemius, tibialis anterior, and gluteus medius) in DN patients (Kwon et al., 2003; Sawacha et al., 2012). The application of gait therapy may thus improve muscles within the kinetic chain that contribute to propulsion in multijoint movements such as walking (Stastny et al., 2016).

**TABLE 1:** Progression of intensity and session duration of the endurance-dominated exercise program in combination with gait therapy across the 12 weeks intervention period.

Week	Sections of each session	Intensity in % of VO_2_max during exercising on a bike ergometer	Section time within each session (minutes)
Weeks 1, 2	Warm-up	50	10
	Main section	55	25
	Cooldown	50	5
Weeks 3, 4	Warm-up	55	10
	Main section	60	30–35
	Cooldown	55	5
Weeks 5, 6	Warm-up	55	10
	Main section	60–65	35–40
	Cooldown	55	5
Weeks 7, 8	Warm-up	60	10
	Main section	65–70	35
	Cooldown	60	5
Weeks 9, 10	Warm-up	60	10
	Main section	70	30
	Cooldown	60	5
Weeks 11, 12	Warm-up	65	10
	Main section	70–75	25
	Cooldown	65	5

*During each week, three exercise sessions were conducted resulting in 36 exercise sessions overall. Warm-up: Gait exercises at slow, habitual, and fast walking speeds, dynamic stretching movements. Main section: Training on a bike ergometer at the above described % VO_2_max intensities and gait exercises including continuous walking (5 min), stair climbing and descending (5 min), tandem walking (5 min), and walking sideways (5 min). Gait training was always conducted prior to endurance training on the bike ergometer. Cooldown: dynamic stretching.*

### Assessment of Maximal Oxygen Uptake (VO_2_max) and the Target Heart Rate Frequency

Maximum oxygen consumption was measured using a standardized graded exercise test performed on a bike ergometer (Warren E. Collins, Braintree, MA, United States). Participants were asked to choose a comfortable pedaling rate equal to or higher than 50 rpm and to maintain that rate throughout the test. After a 2-min warm-up period at 50 W, the test was initiated at an initial power output of 50 W. Increments of 15 W were made every min until exhaustion (Storer et al., 1990). For the assessment of VO_2_max, the PowerCube open-circuit spirometry (PowerCube-Ergospirometer, Germany, Ganshorn) was used to continuously collect and analyze the expired gases (Billinger et al., 2008; Kluding et al., 2015). In addition, heart rate was continuously monitored throughout each stage of the test using the Polar Vantage XL heart rate monitor (Polar Electro, Kempele, Finland). Before the test started, the test equipment was calibrated according to the manufacturer’s recommendations. Rate of perceived exertion was assessed after each stage of the test using Borg’s 6–20 visual analog scale (Borg, 1998). The test was terminated if patients reached a VO_2_max plateau, a respiratory exchange ratio greater than or equal to 1.15, and/or a peak heart rate within 85% of the age-predicted maximal heart rate. The test was stopped if any of the following occurred: angina, dyspnea, fatigue (voluntary exhaustion or inability to maintain a pedaling rate equal or higher than 50 rpm), hypertension (>250 mm Hg systolic or >115 mm Hg diastolic), hypotension, or ischemic electrocardiography abnormalities (Kluding et al., 2015).

### Assessment of Walking Kinetics

A force plate (Bertec Corporation, Columbus, OH, United States) was used to record GRF data during walking at a sampling rate of 1000 Hz. Participants were asked to walk at a constant speed of ∼1.00 m/s over an 18 m walkway. Three practice trials were performed to familiarize the participants with the test before performing five test trials with a 5-min rest between each trial to minimize the effects of fatigue.

Kinetic data were processed as described by Jafarnezhadgero et al. (2019a). GRFs were low pass filtered at 20 Hz (4th order Butterworth filter, zero lag). Specific gait characteristics (heel strike and toe-off) were identified using the Bertec force plate. For this purpose, a 10 N threshold was used to detect the stance phase of the gait cycle. The following dependent variables were extracted from GRF data (Jafarnezhadgero et al., 2019a): First (Fz_*HC*_) and second vertical peak force (Fz_*PO*_). Braking (Fy_*HC*_) and propulsion forces (Fy_*PO*_) were recorded from the anterior–posterior force curve. From the medial-lateral curve, we calculated the positive (lateral) peak (Fx_*HC*_) which occurs right after heel contact. Moreover, we additionally assessed the negative peak which corresponds to the transfer of body mass to the contralateral limb (Fx_*PO*_). GRF amplitudes were normalized to body weight (BW) and reported in %BW. Time to peak (TTP) was defined as the time between the initial heel contact and the corresponding peak of GRF components. The loading rate was defined as the slope between heel contact and Fz_*HC*_ on the vertical force curve. The free moment (FM) of the foot was also computed. Moreover, FM amplitudes were normalized with regards to BW × height. All gait variables were averaged across five trials (Jafarnezhadgero et al., 2019a). For stance phase analysis, GRF data were normalized to 101 data points.

### Assessment of Muscle Activities

A wireless EMG system (EMG Pre-Amplifier, Biometrics Ltd., Nine Mile Point Ind. Est., Newport, United Kingdom) with eight pairs of bipolar Ag/AgCl surface electrodes (25 mm center-to-center distance; input impedance of 100 MO; and common-mode rejection ratio of >110 dB) was used to record the activity of the tibialis anterior (TA), gastrocnemius medialis (Gas-M), biceps femoris (BF), semitendinosus (ST), vastus lateralis (VL), vastus medialis (VM), rectus femoris (RF), and gluteus medius (Glut-M) muscles of the right leg (Hermens et al., 1999). A die-cut medical-grade double-sided adhesive tape (T350, Biometrics Ltd., Nine Mile Point Ind. Est., Newport, United Kingdom) was used to attach the electrodes to the muscle bellies. The raw EMG signals were digitized at 1000 Hz and streamed via Bluetooth to a computer for further analysis. According to the European recommendations for surface EMG (SENIAM), the skin surface was shaved and cleaned with alcohol (70% Ethanol–C2H5OH) over the selected muscles (Hermens et al., 1999). Thereafter, the skin was gently abraded before electrode placement (Hermens et al., 1999). GRF and EMG data were synchronized using Nexus software (Oxford Metrics, Oxford, United Kingdom). For EMG analyses, the gait cycle was divided into the following phases: loading phase (0–20% of the gait cycle), mid-stance (20–47% of the gait cycle), and push off (47–70% of the gait cycle) (Jafarnezhadgero et al., 2020). Using a handheld dynamometer, maximum voluntary isometric contraction (MVIC) was assessed for each recorded muscle to normalize EMG during walking to MVIC. Appendix 1 describes the muscle-specific MVIC tests. All normalization procedures were realized in accordance with recommendations from Besomi et al. (2020). For example, the participants were encouraged to perform the tests at maximal effort (Besomi et al., 2020). Three test trials were conducted with 1–2 min rest periods in-between tests (Besomi et al., 2020). For measuring MVIC, an isometric belt (where the joint is locked) was used (set for zero velocity) (Besomi et al., 2020). This instrument is important to control testing factors that can influence the output and facilitate the production of maximal contraction. The maximum value of the MVIC test was considered for normalization purposes (Besomi et al., 2020). After the 12 weeks intervention program, IG participants were re-evaluated following the same procedures as during the first evaluation. Post-tests spanned a period of 6 days after the last training session to make sure that participants were fully recovered after training (Jafarnezhadgero et al., 2019b). The waiting CON did not receive any exercise program during the study period. We offered the control participants to perform the same exercise program after completion of the post-tests. All participants were asked to abstain from any other type of exercise during the intervention period.

### Statistical Analyses

Normal distribution of data was assessed and confirmed using the Shapiro–Wilk test. Baseline between group differences was analyzed using the independent samples *t*-test. To elucidate the effects of the intervention vs. the waiting control group, a 2 (Group: exercise vs. control) × 2 (Time: pre-test vs. post-test) analysis of variance (ANOVA) with repeated measures was computed. *Post-hoc* analyses were calculated using Bonferroni adjusted paired sample *t*-tests. Additionally, effect sizes were determined by converting partial eta-squared ($\eta_{p}^{2}$) from ANOVA output to Cohen’s d. Within group effect sizes were computed using the following equation: mean difference of pre- and post-tests/pooled standard deviation. According to Cohen, *d* < 0.50 indicate small effects, 0.50 ≤ *d* < 0.80 indicate medium effects, and *d* ≥ 0.80 indicate large effects (Cohen, 2013). Intraclass correlation coefficients (ICC) were calculated for all analyzed variables using pre, post data from the control group (Table 2). In accordance with Koo and Li (2016), test–retest reliability in the form of ICCs was calculated using two-way mixed models (Koo and Li, 2016). ICC values less than 0.5 are indicative of poor reliability, values between 0.5 and 0.75 indicate moderate reliability, values between 0.75 and 0.9 indicate good reliability, and values greater than 0.90 indicate excellent reliability (Koo and Li, 2016). The significance level was set at *p* < 0.05. The statistical analyses were computed using SPSS (version 24, SPSS Inc., 8 Chicago, IL, United States).

**TABLE 2:** Intraclass correlation coefficients (ICC) for all analyzed variables using pre, post data from the waiting control group.

Variables	Component	ICCs
Vo_2_max (ml. kg. min)	–	0.81
Walking stance time (ms)	–	0.84
Vertical ground reaction force (%BW)	Fx_*HC*_ (% BW)	0.78
	Fx_*PO*_ (% BW)	0.79
	Fy_*HC*_ (% BW)	0.84
	Fy_*PO*_ (% BW)	0.87
	Fz_*HC*_ (% BW)	0.92
	F_*ZPO*_ (% BW)	0.90
Time to peak force (ms)	Fz_*HC*_	0.91
Free moment (%BW × height)	Negative FM × 10^–3^	0.81
	Positive FM × 10^–3^	0.83
Loading rate (N/kg/s)	Vertical	0.91
TA (%MVIC)	LR	0.81
	MS	0.79
	PO	0.81
Gas-M (%MVIC)	LR	0.78
	MS	0.84
	PO	0.82
VL (%MVIC)	LR	0.77
	MS	0.80
	PO	0.80
VM (%MVIC)	LR	0.79
	MS	0.76
	PO	0.81
RF (%MVIC)	LR	0.77
	MS	0.74
	PO	0.76
BF (%MVIC)	LR	0.75
	MS	0.79
	PO	0.80
ST (%MVIC)	LR	0.77
	MS	0.79
	PO	0.77
Glut-M (%MVIC)	LR	0.75
	MS	0.80
	PO	0.78

*BW, body weight; VO2max: Peak oxygen uptake; Fx_*HC*_, Peak lateral ground reaction force at heel contact; Fx_*PO*_, Peak medial ground reaction force during push-off; Fy_*HC*_, Braking reaction force; Fy_*PO*_, Propulsion force; Fz_*HC*_; First peak of vertical ground reaction force at heel contact; Fz_*PO*_, vertical ground reaction force during push-off; BW, body weight; TTP, Time to Peak; ms, millisecond; FM, free moment; LR, loading rate; MS, mid-stance; PO, push-off; TA, tibialis anterior; Gas-M, gastrocnemius medialis; VM, vastus medialis; VL, vastus lateralis; BF, biceps femoris; ST, semitendinosus; RF, rectus femoris; Glut-M, gluteus medius; MVIC, maximum voluntary isometric contraction.*

## Results

All participants received treatment as allocated. Adherence rate for both groups were 100%. No training or test related injuries were reported over the course of the study.

Participants’ characteristics are illustrated in Table 3. There were no significant between group baseline differences for demographic, anthropometric, physiological, and biomechanical data (*p* > 0.05).

**TABLE 3:** Group specific baseline data for demographic, anthropometric, physiological, and biomechanical (kinetics, muscle activity) data.

Characteristics	Variables	Component	Waiting Control	Intervention	95% CI	*p*-value
			Mean ± SD	Mean ± SD		
Demographics	Age (years)	–	55.5 ± 5.8	54.1 ± 5.6	−4.3,1.6	0.363
	Body mass index (kg/m2)	–	27.9 ± 4.0	27.4 ± 4.3	−0.8,0.9	0.759
	Gender (male/female), n	–	(20,10), 30	(20,10), 30	–	–
	Smoker	–	20: NO 10: Yes	19: NO 11: Yes	–	–
	Time from first diabetes diagnosis (years)	–	14.2 ± 2.0	14.8 ± 2.8	−0.6,1.9	0.303
	Duration of neuropathy	–	5.8 ± 1.0	6.1 ± 1.5	−0.3,1.0	0.335
Anthropometrics	Waist circumference (cm)	–	97.5 ± 13.0	99.7 ± 13.8	−4.7,9.1	0.530
	Hip circumference (cm)	–	101.5 ± 10.5	95.9 ± 11.1	−11.1,0.0	0.051
	Body fat (%)	–	28.8 ± 3.3	29.4 ± 2.9	−0.9,2.2	0.434
Physiological variables	Vo2max (ml. kg. min)	–	18.1 ± 0.7	18.8 ± 0.8	−0.1,0.6	0.293
	Resting systolic blood pressure (mm Hg)	–	133.5 ± 25.8	127.8 ± 17.7	−17.1,5.7	0.327
	Resting diastolic blood pressure (mm Hg)	–	77.2 ± 10.1	73.4 ± 21.8	−12.6,4.9	0.383
	Resting heart rate (bpm)	–	78.2 ± 12.9	75.0 ± 7.6	−8.6,2.3	0.259
	HbA1c (%)	–	8.8 ± 1.5	9.1 ± 1.7	−0.5,1.1	0.466
	Taking daily insulin	–	23: Yes 7: NO	24: Yes 6: NO	−	−
	Fuzzy score	−	6.0 ± 0.4	5.8 ± 0.9	–0.6,0.1	0.175
Walking stance time (ms)	–	–	578.00 ± 12.45	578.20 ± 9.93	−6.02,5.62	0.945
Kinetics	Vertical ground reaction force (%BW)	FxHC	5.23 ± 2.86	6.09 ± 3.18	−2.4,0.7	0.279
		FxPO	−6.13 ± 3.08	−5.12 ± 3.03	−2.5,0.5	0.207
		FyHC	−14.98 ± 2.82	−14.99 ± 3.58	−1.6,1.6	0.988
		FyPO	13.47 ± 5.60	13.31 ± 2.69	−2.1,2.4	0.891
		FzHC	101.67 ± 18.20	99.85 ± 12.79	−6.3,9.9	0.657
		FZPO	92.01 ± 5.95	92.05 ± 6.58	−3.2,3.3	0.982
	Time to peak force (ms)	FzHC	252.03 ± 26.36	249.30 ± 29.84	−1.8,27.2	0.085
	Free moment (% BW × height)	Negative FM × 10^–3^	−6.79 ± 4.55	−7.144.79	−2.0,2.7	0.770
		Positive FM × 10^–3^	13.75 ± 9.20	15.70 ± 10.70	−7.1,3.2	0.453
	Loading rate (N/kg/s)	Vertical	3.93 ± 0.88	4.05 ± 0.65	−0.5,0.3	0.563
Muscle activities	TA (%MVIC)	LR	46.03 ± 6.5	46.2 ± 15.7	−6.1,6.3	0.976
		MS	13.2 ± 6.4	14.4 ± 3.2	−3.7,1.4	0.387
		PO	13.0 ± 3.1	12.2 ± 4.4	−1.1,2.8	0.413
	Gas-M (%MVIC)	LR	15.7 ± 5.5	15.8 ± 6.4	−3.2,3.0	0.961
		MS	17.9 ± 4.7	17.5 ± 5.0	−2.1,2.8	0.780
		PO	30.7 ± 6.0	29.9 ± 3.5	−1.7,3.3	0.479
	VL (%MVIC)	LR	25.8 ± 12.4	26.3 ± 13.9	−7.3,6.3	0.878
		MS	28.5 ± 10.8	27.9 ± 9.6	−4.7,5.8	0.827
		PO	14.4 ± .9	14.1 ± 5.8	−2.4,3.0	0.830
	VM (%MVIC)	LR	23.6 ± 8.7	24.5 ± 20.9	9.1,7.4	0.840
		MS	15.9 ± 10.4	13.5 ± 6.3	−2.0,6.8	0.285
		PO	15.3 ± 6.7	16.9 ± 5.8	−4.8,1.6	0.319
	RF (%MVIC)	LR	20.6 ± 10.5	22.5 ± 11.0	−7.4,3.7	0.504
		MS	18.9 ± 14.9	19.9 ± 8.9	−7.3,5.3	0.760
		PO	19.4 ± 8.7	20.4 ± 6.3	−4.9,2.9	0.622
	BF (%MVIC)	LR	13.1 ± 12.3	14.1 ± 13.3	−7.6,5.5	0.756
		MS	11.7 ± 7.5	12.5 ± 6.1	−4.3,2.7	0.644
		PO	14.6 ± 8.3	15.5 ± 9.7	−5.6,3.7	0.680
	ST (%MVIC)	LR	16.4 ± 13.6	18.4 ± 10.6	−8.3,4.2	0.520
		MS	13.5 ± 10.6	13.9 ± 8.2	−5.3,4.5	0.864
		PO	14.1 ± 13.5	11.2 ± 9.6	−3.2,8.9	0.347
	Glut-M (%MVIC)	LR	18.0 ± 10.8	17.2 ± 13.1	−5.4,6.9	0.812
		MS	19.0 ± 11.4	22.7 ± 11.5	−9.5,2.2	0.225
		PO	16.5 ± 11.8	16.9 ± 8.8	−5.7,5.0	0.891

*IG, intervention group; CON, control group; BW, body weight; VO2max: Peak oxygen uptake; Fx_*HC*_, Peak lateral ground reaction force at heel contact; Fx_*PO*_, Peak medial ground reaction force during push-off; Fy_*HC*_, Braking reaction force; Fy_*PO*_, Propulsion force; Fz_*HC*_; First peak of vertical ground reaction force at heel contact; Fz_*PO*_, vertical ground reaction force during push-off; BW, body weight; TTP, Time to Peak; ms, millisecond; FM, free moment; LR, loading rate; MS, mid-stance; PO, push-off; TA, tibialis anterior; Gas-M, gastrocnemius medialis; VM, vastus medialis; VL, vastus lateralis; BF, biceps femoris; ST, semitendinosus; RF, rectus femoris; Glut-M, gluteus medius; MVIC, maximum voluntary isometric contraction; SD, standard deviation; CI, confidence interval.*

Table 2 shows test–retest reliability for all analyzed variables using ICCs.

### Maximal Oxygen Uptake (VO_2_max)

Significant group-by-time interactions were found for VO_2_max (*p* < 0.001; *d* = 1.23). *Post-hoc* analyses revealed training induced increases in VO_2_max for IG but not CON (*p* < 0.001; *d* = 1.88) (Table 4).

**TABLE 4:** Group-specific VO2max data before and after training.

Parameter	CON		Δ (%)	95% CI	IG		Δ (%)	95% CI	*p*-value (effect size Cohen’s d)		
	Pre-test	Post-test			Pre-test	Post-test			Main effect: Time	Main effect: Group	Interaction: Group × Time
VO_2_max (ml. kg. min)	18.1 ± 7.7	17.8 ± 0.7	0.5	−0.1,0.0	18.8 ± 0.8	20.5 ± 0.9	9.0	−1.7,−1.4	**<0.001 (1.688)**	**<0.001 (1.250)**	<0.001 (1.226)

*IG, Intervention group; CON, Control group; VO_2_max: maximum oxygen uptake. Bold highlight large effects.*

### Walking Kinetics

**FIGURE 2:**
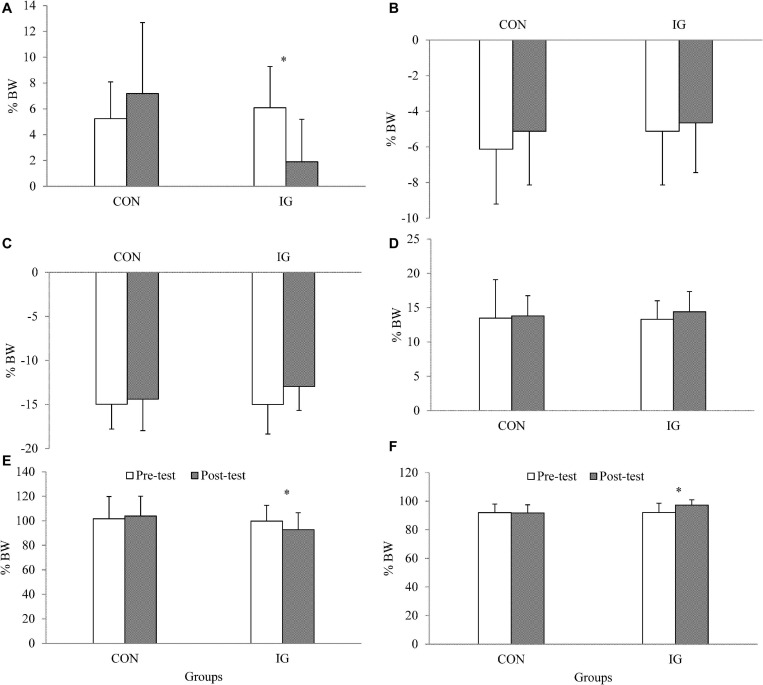
Data are presented as means and standard deviations and illustrate group-specific ground reaction forces during walking at constant speed (1 m/s) before and after training. IG, intervention group; CON, control group; BW, body weight; **(A)** FxHC, peak lateral ground reaction force at heel contact; **(B)** FxPO, peak medial ground reaction force during push-off; **(C)** FyHC, braking reaction force; **(D)** FyPO, propulsion force; **(E)** FzHC; first peak of vertical ground reaction force at heel contact; **(F)** FzPO, vertical ground reaction force during push-off.

For the parameter walking stance time, no significant group-by-time interactions were found (*p* = 0.313; *d* = 0.27) (Table 5). Significant group-by-time interactions were observed for Fx_*HC*_, Fz_*HC*_, and Fz_*PO*_ (*p* < 0.037; *d* = 0.56–1.53). *Post-hoc* analysis revealed decreases in Fx_*HC*_ (*p* < 0.001; *d* = 1.33) and Fz_*HC*_ (*p* = 0.004; *d* = 0.55) and increases in Fz_*PO*_ (*p* < 0.001; *d* = 0.92) in the IG (Figure 2 and Table 5).

**TABLE 5:** Group-specific stance time and kinetic data during walking at constant speed (1 m/s) before and after training.

Parameter	CON				Δ (%)	95% CI	IG				Δ (%)	95% CI	*p*-value (effect size Cohen’s d)		
	Pre-test		Post-test				Pre-test		Post-test				Main effect: Time	Main effect: Group	Interaction: Group × Time
	*M*	SD	*M*	SD			*M*	SD	*M*	SD					
**Walking stance time (ms)**	**578.00**	**12.45**	**577.50**	**8.56**	**0**	**−2.3, 3.3**	**578.20**	**9.93**	**575.70**	**9.34**	**0**	**−1.3, 5.3**	**0.132 (0.403)**	**0.744 (0.090)**	**0.313 (0.271)**
TTPFz_*HC*_ (ms)	262.03	26.36	263.50	26.43	0	−16.2, 13.2	249.30	29.84	259.5	26.62	4	−24.9, 4.5	0.258 (0.30)	0.396 (0.22)	0.092 (0.44)
Negative FM × 10^–3^	−6.79	4.55	−7.60	5.23	11	−6.3, 3.1	−7.14	4.79	−7.82	4.25	9	−1.5, 2.8	0.359 (0.24)	0.753 (0.09)	0.936 (0.00)
Positive FM × 10^–3^	13.75	9.20	15.36	7.98	11	−1.6, 3.2	15.70	10.70	13.27	9.13	−41	−2.2, 7.0	0.800 (0.06)	0.967 (0.00)	0.217 (0.32)
Loading rate (N/BW/s)	3.93	0.88	3.97	0.69	1	−0.4, 0.3	4.05	0.65	3.61	0.67	−11	0.1, 0.7	0.108 (0.42)	0.408 (0.22)	0.053 (0.51)

IG, intervention group; CON, control group; BW, body weight; FM, free moment; Fz_*HC*_; first peak of vertical ground reaction force at heel contact; TTP, time to peak; M, mean; SD, standard deviation; Δ (%), the value of progress calculated as: [(post-test–pre-test)/pre-test]*100; CI, confidence interval. Significant *p*-values were highlighted in bold.

### Muscle Activities

Significant group-by-time interactions were identified for Gas-M activities during push-off, VL activities during loading, VM activities during mid-stance, and Glut-M activities during the loading phase (*p* < 0.044; *d* = 0.54–0.81) (Table 6). *Post-hoc* tests revealed decreases in Gas-M activities during the push-off phase in the IG (*p* < 0.001; *d* = 1.28). In addition, significant increases were found for VL (*p* = 0.001; *d* = 1.08) and Glut-M (*p* = 0.008; *d* = 0.67) activities during loading, and VM activities during mid-stance phase (*p* = 0.001; *d* = 0.86) in IG.

**TABLE 6:** Group-specific normalized muscle activities (% MVIC) during walking at constant speed before and after training.

Muscle		CON				Δ (%)	95% CI	IG				Δ (%)	95% CI	*p*-value (effect size Cohen’s d)		
		Pre-test		Post-test				Pre-test		Post-test				Main effect: Time	Main effect: Group	Interaction: Group × Time
		*M*	SD	*M*	SD			*M*	SD	*M*	SD					
TA	LR	46.3	6.5	45.6	6.7	−1	−2.7,4.2	46.2	15.7	43.2	15.9	−6	−5.4,11.5	0.406 (0.22)	0.568 (0.15)	0.611 (0.12)
	MS	13.2	6.4	13.0	7.8	−1	−3.5,3.9	14.4	3.2	13.7	4.5	−4	−1.6.2.9	0.685 (0.11)	0.381 (0.23)	0.833 (0.06)
	PO	13.0	3.1	13.8	3.6	6	−2.6,1.1	12.2	4.4	13.3	4.0	9	−3.1/1.0	0.199 (0.33)	0.368 (0.23)	0.807 (0.06)
Gas-M	LR	15.7	5.5	14.4	2.8	−8	−1.3,3.9	15.8	6.4	15.4	5.0	2	−2.6,3.4	0.388 (0.23)	0.537 (0.16)	0.634 (0.12)
	MS	17.9	4.7	18.4	5.4	2	−3.5,2.5	17.5	5.0	20.2	6.3	15	−5.8,0.4	0.143 (0.39)	0.419 (0.21)	0.310 (0.27)
	PO	30.7	6.0	29.6	5.2	−3	−1.9,4.0	29.9	3.5	23.6	5.0	−21	3.8,8.7	**<0.001 (1.01)**	**<0.001 (1.00)**	**0.007 (0.72)**
VL	LR	25.8	12.4	25.4	13.5	−1	−6.4,7.1	26.3	13.9	44.8	21.0	70	−28.9,−8.1	**0.004 (0.78)**	**<0.001 (0.98)**	**0.003 (0.81)**
	MS	28.5	10.8	26.8	10.3	−5	−2.3,5.7	27.9	9.6	30.7	10.0	10	−7.5,2.0	0.719 (0.01)	0.450 (0.20)	0.152 (0.38)
	PO	14.4	4.9	13.3	11.2	−7	−2.9,5.0	14.1	5.8	16.8	6.3	19	−6.2,0.8	0.540 (0.15)	0.274 (0.29)	0.154 (0.38)
VM	LR	23.6	8.7	22.2	15.3	−5	−5.6,8.5	24.5	20.9	26.2	15.3	6	−.9.9,6.5	0.960 (0.00)	0.435 (0.70)	0.559 (0.15)
	MS	15.9	10.4	15.1	9.0	−5	−4.3,5.9	13.5	6.3	22.8	14.8	68	−14.8,−3.9	**0.023 (0.6**1)	0.195 (0.30)	**0.007 (0.72)**
	PO	15.3	6.7	15.9	6.5	3	−3.8,2.6	16.9	5.8	16.7	8.5	−1	−3.4,3.8	0.880 (0.00)	0.357 (0.24)	0.747 (0.09)
RF	LR	20.6	10.5	21.3	13.3	3	−4.7,3.3	22.5	11.0	27.8	11.8	23	−10.4,−0.1	0.065 (0.49)	0.110 (0.42)	0.156 (0.37)
	MS	18.9	14.9	19.8	14.8	5	−6.8,5.0	19.9	8.9	25.6	14.1	28	−9.8,−1.5	0.067 (0.49)	0.260 (0.30)	0.180 (0.35)
	PO	19.4	8.7	18.4	9.4	5	−2.9,5.0	20.4	6.3	21.9	11.8	7	−5.0,2.1	0.880 (0.00)	0.269 (0.29)	0.341 (0.25)
BF	LR	13.1	12.3	15.2	10.4	16	−8.8,4.5	14.1	13.3	25.5	12.9	80	−19.0,−3.6	**0.009 (0.71)**	**0.006 (0.74)**	0.069 (0.48)
	MS	11.7	7.5	11.3	8.3	−3	−2.7,3.4	12.5	6.1	12.0	6.5	−4	−2.2,3.2	0.660 (0.11)	0.631 (0.12)	0.945 (0.00)
	PO	14.6	8.3	11.8	13.5	−19	−1.8,7.4	15.5	9.7	19.1	13.9	23	−10.1,3.0	0.844 (0.06)	0.072 (0.48)	0.112 (0.42)
ST	LR	16.4	13.6	16.6	13.7	−1	−7.8,7.4	18.4	10.6	17.5	12.9	−4	−5.0,6.7	0.89 (0.00)	0.518 (0.16)	0.820 (0.06)
	MS	13.5	10.6	11.7	11.9	−5	−3.5,7.2	13.9	8.2	14.7	12.9	5	−6.3,4.9	0.772 (0.063)	0.464 (0.19)	0.501 (0.18)
	PO	14.1	13.5	11.2	8.9	−20	−3.2,9.0	11.2	9.6	7.7	5.1	−31	−0.8,7.9	0.086 (0.45)	0.069 (0.48)	0.867 (0.00)
Glut-M	LR	18.0	10.8	19.1	8.6	6	−6.7.4.4	17.2	13.1	28.0	18.7	62	−18.4,−3.0	**0.013 (0.67)**	0.118 (0.41)	**0.043 (0.54)**
	MS	19.0	11.4	22.4	14.9	17	−11.1,4.3	22.7	11.5	27.1	19.0	20	−12.9,4.1	0.171 (0.32)	0.104 (0.43)	0.858 (0.06)
	PO	16.5	11.8	13.9	13.1	−15	−4.0,9.2	16.9	8.8	22.5	17.5	33	−13.7,2.4	0.546 (0.15)	0.097 (0.44)	0.114 (0.41)

*LR, loading rate; MS, mid-stance; PO, push-off; IG, intervention group; CON, control group; M, mean; SD, standard deviation; Δ (%), the value of progress calculated as: [(post-test–pre-test)/pre-test] × 100; CI, confidence interval; TA, tibialis anterior; Gas-M, gastrocnemius medialis; VM, vastus medialis; VL, vastus lateralis; BF, biceps femoris; ST, semitendinosus; RF, rectus femoris; Glut-M, gluteus medius. Significant *p*-values were highlighted in bold.*

## Discussion

The aim of this study was to evaluate the effects of an endurance-dominated exercise program on VO_2_max and ground reaction forces as well as muscle activities during walking at constant speed in patients with DN.

The main findings of this study can be summarized as follows. The analyses revealed (i) significant increases and large sized effects of the endurance-dominated exercise program on VO_2_max in DN patients; (ii) significant decreases and medium-to-large sized training effects for peak lateral and vertical ground reaction forces during heel contact and a significant increase and medium-to-large sized training effect for peak vertical ground reaction force at push-off; (iii) significant increases and medium-to-large sized training effects for VL and Glut-M activities during the loading phase of walking and VM activities at mid-stance; (iv) significant decreases and large sized training effects for Gas-M activities during walking at push-off.

### Maximal Oxygen Uptake (VO_2_max)

There is evidence in the literature showing that DN patients aged 30–50 years have a significantly lower VO_2_max (∼29%) compared with healthy controls (Regensteiner et al., 1998). Our study participants were aged 45–65 years and had a mean VO_2_max at baseline of 18.1 ml. kg. min (CON) and 18.8 ml. kg. min (IG). According to the American College of Sports Medicine (ACSM), average VO_2_max of healthy males and females aged 46–55 years amounts to 33 and 30 ml. kg. min, respectively (Medicine, 2013). Our participants’ peak aerobic capacity was clearly below these sex-specific norm values. According to a cross-sectional study that reported reference values for peak oxygen uptake during cycle ergometry from a sample of 10,090 participants (6,462 men and 3,628 women), VO_2_max values of the included patients are in the range of the 10th percentile for 69 year old adults (Rapp et al., 2018). These numbers indicate that the capacity of our study participants to perform activities of daily living was most likely limited. Consequently, training programs should aim to enhance DN patients’ maximal oxygen uptake to maintain mobility and the capacity to perform activities of daily living (Nuttamonwarakul et al., 2012). The applied endurance-dominated program resulted in a ∼9% increase in VO_2_max after 12 weeks of training which is in accordance with the literature and confirms our study hypothesis. In fact, Rehman et al. (2017) examined the effects of a 25 week endurance training program including three weekly exercise sessions on VO_2_max in DN patients. After training, the authors observed a statistically significant 8.6% increase in VO_2_max which was similar to our study (Rehman et al., 2017). Exercise-induced improvements in VO_2_max are functionally relevant given that VO_2_max is a strong and independent predictor of all-cause and disease-specific mortality, regardless of sex and race (Harber et al., 2017).

### Walking Kinetics

Diabetic neuropathy patients aged 40–70 years have a significantly lower second peak of vertical ground reaction force (∼3.6%) compared with healthy controls when walking at preferred speed (Akashi et al., 2008). Accordingly, propulsion during walking is reduced which negatively affects walking speed (Damavandi et al., 2012). Walking speed is a simple, valid, and reliable biomarker of mobility status and strongly associated with morbidity and even mortality (Van Kan et al., 2009). Therefore, training-induced increases in the second peak of vertical ground reaction force are warranted to improve walking speed. The applied endurance-dominated exercise program resulted in a 5.6% increase in the second peak of vertical ground reaction force which contributed to a stronger push-off during walking.

Our findings demonstrated exercise-induced decreases in the first vertical and lateral ground reaction forces by 7.1 and 68.8%, respectively. A previous study suggested that the plantar pressure distribution was related to the shear ground reaction force (e.g., lateral ground reaction force) (Savelberg and De Lange, 1999). Therefore, lower first lateral ground reaction forces may be associated a reduced risk of sustaining pressure-related ulceration. However, further studies are needed to verify these findings. Notably, increased impact shocks may constitute biomechanical risk factors for orthopedic injuries such as low back pain, knee osteoarthritis or stress fractures (Radin et al., 1978; Hennig and Lafortune, 1991; Farahpour et al., 2016). The endurance-dominated training program resulted in a decrease in impact shocks which could possibly reduce the injury risk for DN patients due to a lower first vertical ground reaction force amplitude.

Given that there is no other study available in the literature that examined the effects of an endurance-dominated exercise program on walking kinetics in individuals with DN, we decided to compare our findings with studies that investigated the effects of different training programs (e.g., multimodal training) on walking mechanics in DN patients. For instance, Sartor et al. (2014) evaluated the effects of a multimodal exercise program including strength and flexibility exercises on peak pressure of six different foot areas, ankle kinetics and kinematics during walking in DN patients aged 45–65 years. After training, the authors observed improved eccentric control of forefoot contact in the form of a decrease in the ankle extensor moment and a concomitant increase in ankle dorsiflexion range of motion. This may have resulted in improved shock absorption capacity (lower first peak of vertical ground reaction force) during walking as indicated in this study. In another study, El-Refay and Ali (2013) evaluated the effects of strength training for the toe flexors/extensors along with balance/gait training on gait function of DN patients with a mean age of ∼57 years. The intervention compared to the control group showed improved walking speed, cadence and ankle range of motion after training. Researchers previously speculated that the training-induced improvements could be due to improved macro and microvascular factors (Higashi et al., 1999; El-Sayed et al., 2005). In other words, vascular adaptations caused by exercise may facilitate blood flow to peripheral nerves, which may again result in improved gait function (Melese et al., 2020). A recent systematic review of randomized controlled trials evaluated the impact of exercise therapy (i.e., strength training, balance/gait training) on gait function in DN patients and demonstrated improved walking speed, cadence, and stride length in DN patients compared with controls (Melese et al., 2020). The present study revealed exercise-induced decreases in the first vertical and lateral ground reaction forces which are functionally relevant given that the first vertical and lateral ground reaction forces are predictors of shock absorption and foot pronation (Damavandi et al., 2012).

### Muscle Activities

It has previously been reported that DN patients’ muscle activities during walking are significantly lower for TA, Gas-M, and VL compared with healthy controls (Kwon et al., 2003; Sacco and Amadio, 2003). Therefore, training-induced increases in lower limbs muscle activities during walking are warranted because they improve propulsion and thus walking speed, which has previously been denoted as the sixth vital sign (Fritz and Lusardi, 2009). When taking our findings and the results of previous studies (Kwon et al., 2003; Sacco and Amadio, 2003) together, it can be hypothesized that the applied endurance-dominated training program may have enabled participating patients to walk more efficiently.

In general, our results on the effects of an endurance-dominated exercise program on lower limbs muscle activities in individuals with moderate DN are partially in agreement with findings from the literature (Ahmad et al., 2020). Given that there is no other study available that examined the effects of an endurance-dominated exercise program on lower limbs muscle activities during walking in DN patients, we compare our findings with studies that investigated the effects of different training programs on lower limbs muscle activities during walking in DN patients. For instance Ahmad et al. (2020) examined the effects of an 8 week combined balance (e.g., sit to stand, wobble board exercises, one legged stance, heel, and toe raise) and gait training program (e.g., normal walk, tandem walk, backward walk) including three weekly exercise sessions on activation of lower limbs and multifidus muscles during treadmill walking at self-paced speed in DN patients aged 45–75 years. In contrast to our results, these authors observed a statistically significant increase in Gas-M activity (18.8%) after training (Ahmad et al., 2020). The discrepancy in outcome might be due to different walking speed conditions [self-paced walking speed in the study of Ahmad et al. (2020) vs. constants walking speed in our study] in both studies. In our study, training resulted in significant increases in VL (18.5%) and Glut-M (10.8%) activities during the loading phase of walking and VM (9.3%) activities at mid-stance. These findings imply that the intervention program facilitates shock absorption during the initial contact phase of walking as indicated by increased VL activity (Liikavainio et al., 2010). There is preliminary evidence in the literature (Duval et al., 2010; Chuter and de Jonge, 2012) showing that weakness of the Glut-M when functioning as a hip abductor may be related to an increased risk of sustaining injuries due to excessive subtalar pronation. During walking, the Glut-M contracts prior to and during heel strike to maintain the proper position of the hip, femur, knee, tibia, and foot. Weakness of the Glut-M may result in hip adduction which again causes the femur, knee, and tibia to rotate inward (Bellchamber and van den Bogert, 2000; Bird et al., 2003; Chuter and de Jonge, 2012). This excessive inward rotation of the leg causes an increase in foot pronation. However, shank muscles (e.g., tibialis posterior) that are responsible for the control of foot pronation are not strong enough to counteract these forces from the hip and lower leg (Jafarnezhadgero et al., 2019a). As a consequence, over-pronation sets in which may result in an increased risk of sustaining injuries (Duval et al., 2010; Chuter and de Jonge, 2012).

This study revealed training-induced increases in Glut-M activity during the loading phase of walking and VM activity at mid-stance in DN patients. Previous studies have demonstrated that VL and VM muscles contract in concentric mode as the knee extends from mid-stance to push-off during walking (Mann and Hagy, 1980; Mann et al., 1986). We observed larger VM activities during mid-stance after training.

This study has some methodological limitations that need to be considered. First, we collected kinetic and electromyographic but not kinematic data. Future studies should include the assessment of kinematic data to perform a comprehensive biomechanical analysis. Second, this study was conducted with patients aged 45–65 years with moderate DN. Therefore, we cannot translate our findings to different age and/or patient groups. Third, we did not use loaded walking and step up exercises in our gait therapy protocol. In future studies, researchers should consider to include loaded walking and step up exercises in the gait therapy protocol because this may have an additional muscle strengthening effect (Stastny et al., 2015). Fourth, in this study we investigated diabetic patients with neuropathy only. Future studies may additionally enroll diabetic patients free of neuropathy to examine whether the effects are similar compared to this study. Fifth, in this study we only examined the stance phase of gait. The swing phase could also be of interest when assessing muscle activities during walking which is an open research question for future studies.

## Conclusion

This study showed that an endurance-dominated exercise program has the potential to improve VO2max and diabetes-related abnormal gait in patients with moderate DN. The observed decreases in peak vertical ground reaction force during the heel contact of walking may be due to increased activity of VL and Glut-M during the loading phase. Accordingly, we recommend to implement endurance-dominated exercise programs in type 2 diabetic patients because it is feasible, safe and effective to enhance aerobic capacity and gait characteristics. Future studies may consider to additionally include muscle strengthening exercises in the therapy protocol.

## Data Availability Statement

The datasets presented in this study can be found in online repositories. The names of the repository/repositories and accession number(s) can be found in the article/Supplementary Material.

## Ethics Statement

The studies involving human participants were reviewed and approved by Ardabil University of Medical Sciences. The patients/participants provided their written informed consent to participate in this study.

## Author Contributions

AJ contributed to the editorial input, study design, analysis, and manuscript draft. EM contributed to the study design, data collection, and analysis. UG contributed to the concept, study design, and editorial supervision. All authors contributed to the article and approved the submitted version.

## Conflict of Interest

The authors declare that the research was conducted in the absence of any commercial or financial relationships that could be construed as a potential conflict of interest.

## Footnotes

1 http://www.gpower.hhu.de/
