# Supplementary material for: An Endurance-Dominated Exercise Program Improves Maximum Oxygen Consumption, Ground Reaction Forces, and Muscle Activities in Patients With Moderate Diabetic Neuropathy
Source: Front Physiol. 2021 Mar 18;12:654755. doi: 10.3389/fphys.2021.654755 (PMC8044992; doi:10.3389/fphys.2021.654755)
Supplement: Supplementary file 1 [file Table_1.DOCX]

Appendix 1. Tests for the assessment of maximum voluntary isometric contraction

| Muscles | Test protocol |
| --- | --- |
| TA | In seated position on a chair with backrest, with 90° hip knee and ankle joint ﬂexion. Participants were asked to activate the TA at maximal eﬀort against resistance. |
| Gas-M | In seated position on the examination table with the hip ﬂexed by 90° and the knee in extension and ankle in neutral position (90°). Participants activated their plantar flexors at maximal effort against resistance. |
| BF | In seated position on a chair with hip and knees ﬂexed at 90°. Participants activated the hamstring muscles at maximal effort against resistance. |
| ST | In seated position on a chair with hip and knees ﬂexed at 90°. Participants maximally activated their knee flexors against resistance. |
| VL | In seated position on a chair with hip and knees ﬂexed at 90°. Participants maximally activated their knee extensors against resistance. |
| VM | In seated position on a chair with hip and knees ﬂexed at 90°. Participants maximally activated their knee extensors against resistance. |
| RF | In seated position on a chair with hip and knees ﬂexed at 90°. Participants maximally activated their knee extensors against resistance. |
| Glut-M | In standing position, participants maximally activated their hip abductors against resistance. |

Notes: TA, tibialis anterior; Gas-M, gastrocnemius medialis; BF, biceps femoris; ST, semitendinosus; VL, vastus lateralis; VM, vastus medialis; RF, rectus femoris; Glut-M, gluteus medius.
